# Tubo-ovarian abscess after vaginal delivery: A case report and review of current literature

**DOI:** 10.1016/j.crwh.2023.e00526

**Published:** 2023-07-04

**Authors:** Ruairí Floyd, Breffini Anglim

**Affiliations:** Obstetrics & Gynaecology Department, The Coombe Hospital, Dublin, Ireland

**Keywords:** Pelvic inflammatory disease, Tubo-ovarian abscess, Endometrioma, Puerperal sepsis, Case report

## Abstract

Tubo-ovarian abscesses in pregnancy and the post-partum period are extremely rare. We report a case of a 31-year-old woman who presented with an acute abdomen and sepsis in the post-partum period with a background of a large endometrioma diagnosed prior to conception. Exploratory laparoscopy revealed a ruptured tubo-ovarian abscess which was surgically drained and then treated with intravenous antibiotics. This report is seemingly unique in presenting the development of antenatal endometrioma into a tubo-ovarian abscess and an unusual differential for abdominal pain to consider in the immediate postpartum period.

## Highlights

• An endometrioma rarely develops into a tubo-ovarian abscess.
• The antimicrobial regimen includes doxycycline, metronidazole and a cephalosporin.
• 70% of cases will respond to conservative management.
• Sepsis, poor antibiotic response or peritonitis is an indication for surgical intervention.

## 1 Introduction

Tubo-ovarian abscess (TOA) in pregnancy and the postpartum period is extremely rare. Development of a TOA in the puerperium is extremely uncommon due to the natural barriers to ascending infection, the most common pathophysiology for development of pelvic inflammatory disease (PID) postpartum, during this period [1]. Potential pathways of ascending infection include haematogenous and lymphatic spread of infection, formation of infection in an underlying ovarian cyst, and recurrence of an existing underlying infection [2]. Risk factors for TOA include underlying PID, previous abdominal surgery, congenital anomalies of the genital tract, male partner with an active sexually transmitted infection (STI) and fertility treatments [1,2]. Endometriosis is an often overlooked risk factor for the development of TOA [[2], [3], [4], [5], [6], [7]]. We report a case of a 31-year-old woman who presented with severe abdominal pain in the postpartum period with a background of a large endometrioma. This report is one of the few reports in the literature of formation of a TOA in an existing endometrioma and an unusual differential for abdominal pain to consider in the immediate postpartum period.

## 2 Case Presentation

A 31-year-old woman (gravida 2, para 1) presented to the emergency department four weeks following spontaneous vaginal delivery with acute onset, sharp, generalised abdominal pain which radiated to the right iliac fossa. She was diagnosed with a right-sided endometrioma prior to pregnancy.

**Fig. 1:**
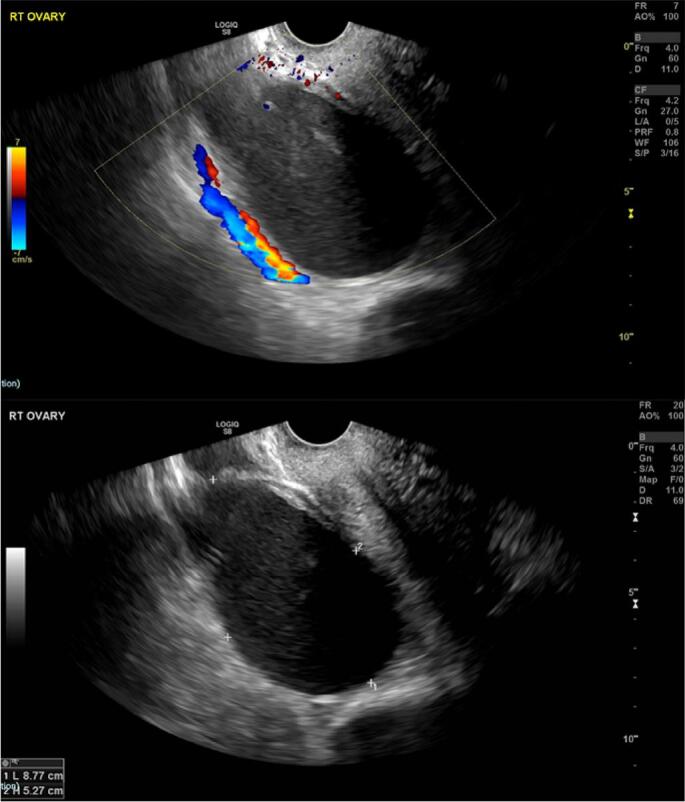
Follow up antenatal ultrasound which showed an increase in size to 9 cm at 4 month interval scan.

**Fig. 2:**
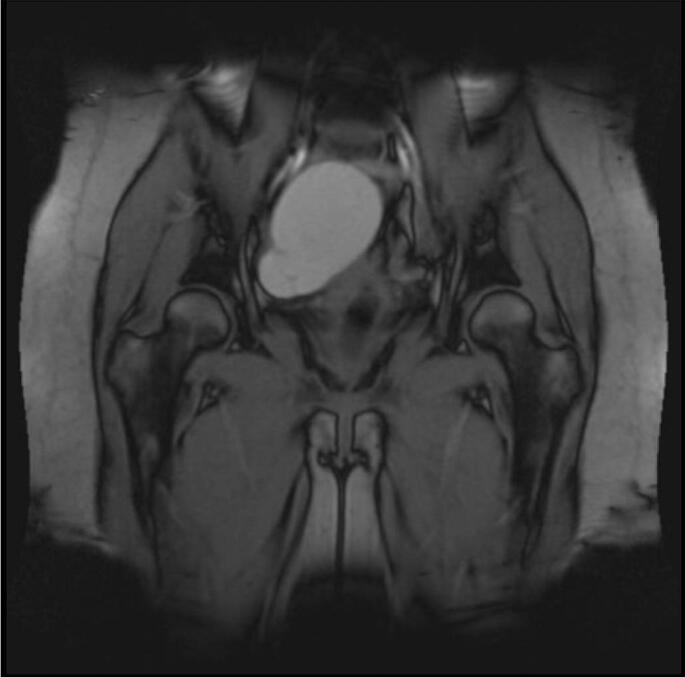
Follow up MRI recommended following increased size of the ovarian cyst which showed the cyst had homogenous hyperintense signal consistent with an endometrioma.

Her past medical history was significant for hypothyroidism, iron deficiency anaemia, anxiety and an appendicectomy. She was a non-smoker, did not consume alcohol and denied any history of intravenous drug abuse or pelvic inflammatory disease. An endometrioma was diagnosed 18 months prior to pregnancy on the basis of ultrasound findings of a hypoechoic cyst with heterogenous echotexture containing diffuse areas of low echogenicity. This was initially 6 cm in size and had increased to 9 cm on four-month follow-up (Fig. 1). It remained unchanged in size 6 months later and was reported to have a homogenous hyperintense signal intensity on T1-weighted images on magnetic resonance imaging (Fig. 2). The option of ovarian cystectomy was discussed with the patient antenatally but she opted not to proceed with surgical management.

She conceived spontaneously following a first-trimester miscarriage eight months previously. She complained of intermittent pain in the right iliac fossa during her pregnancy which had also been present prior to pregnancy. She was diagnosed with COVID-19 at 22 weeks of gestation but otherwise had an uncomplicated pregnancy. She was induced at 39 + 2 for reduced foetal movements and had a spontaneous vaginal delivery with no history of prolonged rupture of membranes. She had a manual removal of placenta and 24 h of intravenous antibiotics (co-amoxiclav) following delivery. Her postpartum period was uneventful and she was discharged 2 days postnatally.

She reported fevers, chills, myalgia and upper respiratory tract symptoms which started 3 days prior to her presenation to the emergency department. She reported an acute-onset sharp pain which woke her from her sleep 3 h prior to presenting to the emergency department. This was associated with two episodes of vomiting and was exacerbated with movement. She reported mild constipation but no urinary symptoms or malodorous lochia. On examination she was pyrexial with a mild tachycardia and was otherwise vitally stable. She had tenderness noted in the right iliac fossa and associated guarding. She had no renal angle tenderness. Speculum exam showed no vaginal discharge and a high vaginal swab was taken. Bimanual examination revealed bilateral adnexal tenderness which was more marked on the right side, with a palpable right adnexal mass. A bedside ultrasound scan showed a hypoechoic right-sided mass measuring 8x6cm. Laboratory studies showed raised inflammatory markers with a raised white cell count of 17.2 × 10^9^/L, neutrophilia of 15.4 × 10^9^/L, cross-reactive protein (CRP) of 260 mg/L and a normal venous gas sample with a lactate of 0.82. Intravenous antibiotics (co-amoxiclav 1.2 g TDS) were commenced. Due to suspected ovarian torsion, an emergency laparoscopy was performed.

**Fig. 3:**
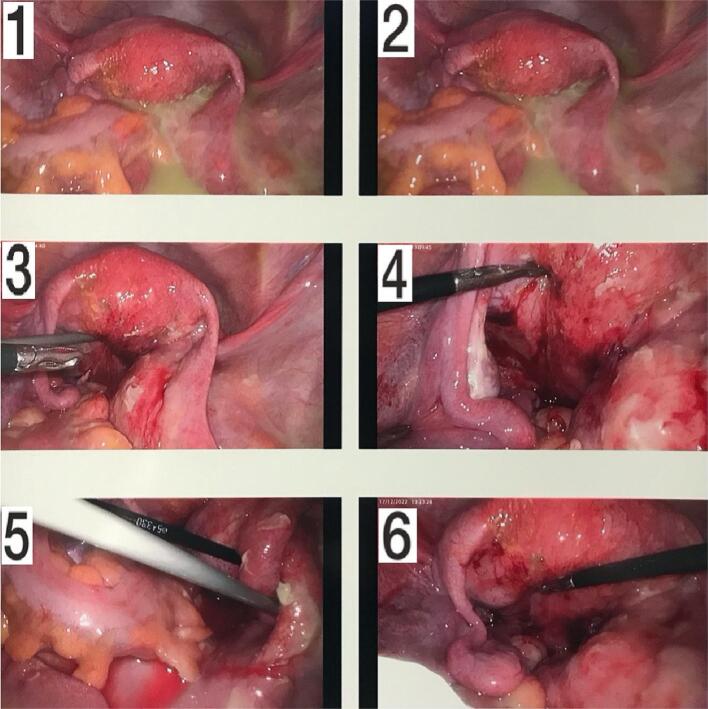
Image 1–2 display purulent exudate in the pelvic cavity. Image 3–6 show a large right sided ovarian abscess, oedematous right fallopian tube with a normal left ovary and evidence of gross salpingitis in the left fallopian tube. Filmy adhesions were present between the left ovary and posterior wall of the uterus, as seen.

Operative findings revealed purulent exudate throughout the pelvis with a large right-sided ovarian abscess, oedematous right fallopian tube with a normal left ovary and gross appearance of acute salpingitis of the left fallopian tube (Fig. 3). The right ovary was adherent to the posterior uterus in the pouch of Douglas. This was separated from the uterus using blunt dissection. There were filmy adhesions between the left ovary and posterior wall of the uterus. The patient underwent laparoscopic drainage of the right tubo-ovarian abscess and thorough peritoneal lavage was performed.

Post-operatively, she was commenced on intravenous ceftriaxone 2 g OD in addition to co-amoxiclav 1.2 g TDS, as recommended by microbiology. The high vaginal swab taken on admission had moderate growth of *Escherichia coli* and *Streptococcus milleri*. Peritoneal fluid showed no significant growth on extended culture. Co-amoxiclav was discontinued according to the microbial sensitivity. COVID-19 and influenza swabs were negative. Her inflammatory markers trended downwards (CRP 359 mg/L preoperatively to 160 mg/L, 146 mg/L, 120 mg/L and 68 mg/L on days 1–5 postoperatively, respectively) and she was discharged on postoperative day 5 with a seven-day course of oral cefalexin. She had a follow-up pelvic ultrasound scan 6 weeks postoperatively which showed complete resolution of the TOA. She had recovered well with no issues at her six-week follow-up and was discharged.

## 3 Discussion

PID and TOA are rare in the antenatal and postpartum period and this case report highlights signs, symptoms and risk factors to consider when a patient presents with signs of sepsis and an acute abdomen in the puerperium. PID is a bacterial infection of the upper genital tract. The most common causative pathogens are *Neisseria gonorrhoeae* and *Chlamydia trachomatis* and less commonly *Mycoplasma genitalium* and enteric pathogens such as *Escherichia coli*, among others [8]. Complications of PID include chronic abdominal pain, dyspareunia, peritonitis, abdominal adhesions, extra-pelvic involvement, increased risk of ectopic pregnancy and tubal infertility [8]. A single episode of PID can significantly increase the risk of tubal infertility, and subsequent episodes of PID further increase this risk [12]. Development of PID and tubo-ovarian abscess in pregnancy is rare due to the natural barriers of pregnancy, including the cervical mucous plus and intact membranes which act to prevent ascending infection [1]. However, it can be associated with sepsis, resulting in subsequent (potential pre-term) delivery, perinatal morbidity and mortality, and pregnancy loss [13]. The main risk factors for development of PID in pregnancy include a history of PID or an STI, fertility treatments or genital anatomical anomalies or pelvic surgery [13]. There are few reports of postpartum PID or tubo-ovarian abscess in the literature [14].

This case report describes an unusual case of postpartum development of an existing endometrioma into a TOA. Risk factors for a tubo-ovarian abscess include PID or previous STI, previous abdomino-pelvic surgery, anatomical anomalies of the genital tract, having a male partner with active STI and fertility treatments, particularly if egg retrieval was undertaken [1,2]. Rarely, endometriomas have been shown to have the potential to develop into a tubo-ovarian abscess [[2], [3], [4], [5], [6], [7]]. The management of known endometriomas in the pre-conception period is unclear as limited evidence exists. Carrying out an ovarian cystectomy of a pre-existing endometrioma prior to conception should be a carefully balanced decision. Factors to be considered include patient symptom severity, existing infertility due to endometriosis, risk of ovarian torsion with increasing size of the endometrioma, risk of damaging ovarian tissue during cystectomy and the effect on ovarian reserve and subfertility postoperatively. Surgical intervention with cystectomy or ablation in the setting of endometriomas has been shown to reduce ovarian reserve, evidenced by a reduction of anti-Mullerian hormone (AMH) [15]. Ovarian cystectomy is potentially more effective than ablation as regards recurrence, pain and fertility [15]. Consideration should also be given to measuring preoperative AMH and avoidance of bipolar instruments to reduce the risk to damaging the ovarian cortex [15].

The above case report highlights an unusual case of tubo-ovarian abscess as an identified cause of acute abdominal pain, fever and vomiting in the postpartum period. Timely presentation and diagnosis should be paramount to allow for initiation of urgent management. The most common presenting signs and symptoms include abdominal pain, fever, night sweats, vaginal discharge, vomiting, loose stools and urinary retention [1,4,5,14,16]. The treatment most commonly employed is an antimicrobial regimen including doxycycline, metronidazole and a cephalosporin [9,10]. Approximately 70% of cases will respond to this conservative management [17]. Surgical intervention is generally not employed unless in the setting of septic shock, failed antimicrobial therapy or peritonitis due to a suspected ruptured abscess [9,11]. Surgical intervention allows for an accurate diagnosis to be made and timely treatment with minimal complications and tubal destruction [18,19]. Larger TOA (>10 cm) are more likely (60%) to require surgical intervention [17]. Surgical intervention has the potential to allow for a more effective antimicrobial response with initial source control, and consequentially shortened hospital stay and decreased future risk of tubal infertility [18,19]. One review suggested that there is minimal clear guidance on the management of tubo-ovarian abscess but suggested that intravenous antibiotics alongside drainage of abscess with interventional radiology may be considered if the tubo-ovarian abscess is <5 cm, whereas laparoscopic drainage is preferable if the TOA is >5 cm [18].

This case highlights the severe and rare complication of development of a longstanding endometrioma into a tubo-ovarian abscess in the postpartum period. Appropriate investigations were carried out with prompt surgical intervention, drainage of the TOA and tailored antibiotic therapy leading to excellent clinical improvement.
